# Metronidazole Treatment Failure and Persistent BV Lead to Increased Frequencies of Activated T- and Dendritic-Cell Subsets

**DOI:** 10.3390/microorganisms11112643

**Published:** 2023-10-27

**Authors:** Wenkosi Perez Qulu, Gugulethu Mzobe, Andile Mtshali, Marothi Peter Letsoalo, Farzana Osman, James Emmanuel San, Asavela Olona Kama, Nigel Garrett, Adrian Mindel, Anne Rompalo, Lenine J. P. Liebenberg, Derseree Archary, Aida Sivro, Sinaye Ngcapu

**Affiliations:** 1 Centre for the AIDS Programme of Research in South Africa (CAPRISA), Durban 4001, South Africa; wenkosi.qulu@caprisa.org (W.P.Q.); gugulethu.mzobe@caprisa.org (G.M.); andile.mtshali@caprisa.org (A.M.); marothi.letsoalo@caprisa.org (M.P.L.); farzana.osman@caprisa.org (F.O.); asavela.kama@caprisa.org (A.O.K.); nigel.garrett@caprisa.org (N.G.); adrian.mindel@gmail.com (A.M.); lenine.liebenberg@caprisa.org (L.J.P.L.); desh.archary@caprisa.org (D.A.); aida.sivro@caprisa.org (A.S.); 2 Department of Medical Microbiology, University of KwaZulu-Natal, Durban 4001, South Africa; 3 KwaZulu-Natal Research Innovation and Sequencing Platform, Nelson R Mandela School of Medicine, University of KwaZulu-Natal, Durban 4001, South Africa; sanemmanueljames@gmail.com; 4 Discipline of Public Health Medicine, University of KwaZulu-Natal, Durban 4001, South Africa; 5 Department of Gynecology and Obstetrics, Johns Hopkins University, Baltimore, MD 21287, USA; arompalo@jhmi.edu; 6 Centre for Epidemic Response and Innovation (CERI), Stellenbosch 7600, South Africa; 7 JC Wilt Infectious Disease Research Centre, National Microbiology Laboratory, Public Health Agency of Canada, Winnipeg, MB R3E 3L5, Canada; 8 Department of Medical Microbiology and Infectious Diseases, University of Manitoba, Winnipeg, MB R3E 3L5, Canada

**Keywords:** vaginal microbiota, genital immune cells, BV treatment

## Abstract

Metronidazole (MDZ) treatment failure and bacterial vaginosis (BV) recurrence rates are high among African women. This cohort study identified genital immune parameters associated with treatment response by comparing vaginal microbiota and immune cell frequencies in endocervical cytobrushes obtained from 32 South African women with symptomatic BV pre- and post-metronidazole treatment. Cervical T- and dendritic-cell subsets were phenotyped using multiparameter flow cytometry and the composition of vaginal microbial communities was characterized using 16S rRNA gene sequencing. MDZ treatment led to a modest decrease in the relative abundance of BV-associated bacteria, but colonization with *Lactobacillus* species (other than *L. iners*) was rare. At 6 and 12 weeks, MDZ-treated women had a significant increase in the frequencies of CCR5+ CD4+ T cells and plasmacytoid dendritic cells compared to the pre-treatment timepoint. In addition, MDZ non-responders had significantly higher frequencies of activated CD4 T cells and monocytes compared to MDZ responders. We conclude that MDZ treatment failure was characterized by an increased expression of activated T- and dendritic-cell subsets that may enhance HIV susceptibility. These data suggest the need to further assess the long-term impact of MDZ treatment on mucosal immune response and the vaginal microbiota.

## 1. Introduction

Bacterial vaginosis (BV) is a polymicrobial condition in the female genital tract (FGT) that can present clinically with vaginal discharge, a fish-like odor, vaginal discomfort, and/or urinary symptoms, and is microbiologically characterized by a lack of beneficial *Lactobacillus* species and by colonization with a diverse spectrum of primarily anaerobic bacteria [1]. Symptomatic BV is usually treated with either oral or topical metronidazole (MDZ) or clindamycin as the standard of care, or with emerging, experimental alternatives including antiseptic treatments, pro- and pre-biotics, and vaginal microbiome transplantation [2,3,4,5,6]. BV treatment leads to a modest reduction in bacterial abundances of BV-associated bacteria (BVAB), but re-colonization with *Lactobacillus* species (other than *L. iners*) is often slow or improbable [7,8,9]. BV recurrence rates following treatment are high and can reach up to 60% within six months of treatment [5,10,11,12,13], suggesting that the effects of BV on normal immune function may manifest over time.

BV has been associated with increased levels of genital inflammation, an important contributing factor to a wide variety of adverse sexual and reproductive outcomes, including an increased risk of preterm birth, cervical dysplasia, miscarriage, and sexually transmitted infections (STIs, including HIV) [14,15,16,17]. In addition, diverse microbial communities reminiscent of bacterial communities found to be associated with BV are closely associated with increased inflammatory mediators and immune cells in the FGT [9,14,15,18]. *Gardnerella vaginalis* increases the concentrations of toll-like receptor (TLR) ligands and pro-inflammatory cytokines, including Interleukin (IL)-1α, IL-1β, IL-6, IL-8, tumor necrosis factor (TNF)-α, and TNF-β, in the FGT [14,18,19,20,21]. Diverse microbial communities dominated by *G. vaginalis* were closely associated with increased levels of IL-17-inducing cytokines (IL-23 and IL-1β) and an increased HIV risk, likely by increasing the mucosal CD4+ T helper 17 (Th17)-cell frequency and immune cell activation [14,15]. Anahtar et al. (2015) detected increased numbers of mucosal CCR5+ CD4+ T cells in mice that received *Prevotella bivia* compared to those intravaginally inoculated with *L. crispatus*. Furthermore, highly diverse microbial communities were directly correlated with increased genital pro-inflammatory cytokine concentrations. This mechanism is likely through an enhanced stimulation of cognate TLR4 and activation of the nuclear factor kappa B (NF-κB) signaling pathway, leading to an increased activation of CCR5+ CD4+ T cells in the female genital tract [14]. Studies suggest that BVAB induces an inflammatory response through the increased production of lipopolysaccharides (LPS), resulting in the activation of the NF–κB pathway by binding to TLR4 and CD14 on genital epithelial cells, monocytes, and macrophages [22,23,24]. While pro-inflammatory cytokine classes are generally upregulated in BV, some studies have shown that certain chemokines (IP10 and monokine induced by gamma-interferon (MIG), in particular) may be downregulated [9,18,25,26]. The hypothesis is that BVAB specifically downregulates chemokines (e.g., interferon-γ-inducible protein (IP)-10 and MIG) that bind to CXCR3 to avoid immune responses from T and other immune cells that express the CXCR3 receptor. In contrast, *Lactobacillus* spp. have been shown to modulate cytokine release by monocyte-macrophages through enhancing the negative regulators of the NF–κB pathway in vitro [27]. *Lactobacillus* spp. have been associated with decreased inflammatory mediator concentrations in the FGT and a reduced inflammatory response to bacterial pathogens in vitro [20,28]. Similarly, *Lactobacillus* spp. significantly suppressed IL-6 and IL-8 production in vaginal epithelial (VK2) cells stimulated with *G. vaginalis* [29]. 

Although studies have demonstrated that vaginal microbiota modulates cytokine and cellular immune response signatures of HIV risk, it remains unclear whether MDZ treatment induces changes in the mucosal immune environment. The relationship between the BV resolution (including treatment failure) and the mucosal immune response remains undefined. To address this research gap, we assessed the composition of vaginal microbiota and cell phenotypes in endocervical cytobrushes of women pre- and post-treatment for BV, including those of women with BV resolution or persistence. We hypothesized that an MDZ treatment and an increase in the dominance of *Lactobacillus* species reduces the mucosal immune response frequency and activation. We further posited that treatment failure or recurrent BV correlates with a more diverse vaginal microbiota that leads to a high number of activated HIV-targeted cells in the FGT.

## 2. Materials and Methods

### 2.1. Study Design, Participants and Specimen Collection

For this ex vivo study, we used endocervical cytobrushes from 32 women with a laboratory-diagnosed STI and/or BV-intermediate or -positive enrolled in the CAPRISA 083 cohort study. The study aimed at reducing STIs in women by enhancing management packages for targeted STI care, ensuring that the individual is cured, and reducing the risk of reinfection using expedited partner therapy [9,30]. Women who were pregnant, living with HIV, or who had received antibiotic treatment within the last 7 days were excluded from the study. Participants diagnosed with *Chlamydia trachomatis* (treatment: azithromycin 1 g oral), *Neisseria gonorrhoeae* (ceftriaxone 250 mg intramuscular and azithromycin 1 g oral), *Trichomonas vaginalis* (metronidazole 2 g oral), Nugent score ≥ 4 (metronidazole 2 g oral), or candidiasis (clotrimazole 500 mg pessary and clotrimazole 1% cream) were asked to return after 6-weeks and 12-weeks post-treatment for follow-up examinations and provided genital specimens for further assays. The protocol for this study was approved by the Ethics Review Committee of the University of KwaZulu-Natal (BREC number: BE303/17). 

### 2.2. BV Classification Using Nugent Score

Vaginal swabs were scored using Nugent criteria and classified as normal (<4), intermediate (4–6), and Nugent-BV (≥7). For the purpose of this study, BV was classified as Nugent score ≥ 4. Women who had Nugent score ≥ 4 at baseline and 6-weeks post-treatment, but had a Nugent score < 4 at 12-weeks post-treatment visit were considered “BV cleared”; while women who had Nugent score ≥ 4 at baseline and all consecutive visits were considered “BV persistence”. Those who had Nugent score < 4 at 6-weeks post-treatment, but a Nugent score that had increased to >4 at 12-weeks post-treatment were classified as having “BV recurrence”.

### 2.3. 16S rRNA Gene Amplicon Sequencing and Upstream Analysis

Genomic DNA was extracted from each vaginal swab using the PowerSoil DNA Isolation kit (Qiagen, Hilden, Germany), and a fragment of the 16S rRNA gene spanning the V3–V4 variable region was PCR amplified using primers 319F (forward) and 806R (reverse), as described previously [9]. Libraries were constructed using ~120 pooled samples that were sequenced on the Illumina MiSeq platform (paired-end sequence reads with v3 chemistry). The ‘DADA2′ R package was used to perform quality-based filtering and trimming, inference of amplicon sequence variants (ASVs), chimera removal, and taxonomy assignment. Taxonomic classification was performed using the SILVA ribosomal RNA database. The ASVs for several key genera (e.g., *Lactobacillus*, *Prevotella*, *Sneathia*, *Mobiluncus*) were classified to the species level using speciateIT (version 1.0, http://ravel-lab.org/speciateIT, accessed on 10 December 2020). Samples were assigned to 4 community state types using VALENCIA (VAginaL community state typE Nearest CentroId clAssifier), a nearest centroid classification algorithm [31]. The reference centroids are available at github.com/ravel-lab/VALENCIA (accessed on 10 December 2020). The ASV table, taxonomy table, and metadata were integrated into a single phyloseq object using the ‘phyloseq’ R package for further downstream analysis and visualization.

### 2.4. Cytokine and Chemokine Measurement

CAPRISA 083 cervicovaginal SoftCup supernatants collected at matching time points with vaginal swabs were used to measure concentrations of 48 cytokines/chemokines using Bio-Plex Pro-Human Cytokine Group I (27-Plex Panel) and Group II (21-Plex Panel) kits (Bio-Rad Laboratories, Hercules, CA, USA), as previously reported [9]. The panel included chemokines, pro-inflammatory cytokines, adaptive, growth factors, and the following anti-inflammatory cytokines: IL-1β, IL-1Rα, IL-2, IL-4, IL-5, IL-6, IL-7, IL-8, IL-9, IL-10, IL-12p70, IL-12p40, IL-16, IL-18, IL-1α, IL-2RA, IL-3, IL-13, IL-15, IL-17, basic fibroblast growth factor (FGF-basic), cutaneous T-cell attracting chemokine (CTACK), Eotaxin, granulocyte colony-stimulating factor (G-CSF), GM–CSF, GRO-α, hepatocyte growth factor (HGF), Interferon (IFN)-γ, IFN-α2, IP-10, leukemia inhibitory factor (LIF), monocyte chemoattractant protein (MCP)-1, MCP-3, macrophage colony-stimulating factor (M-CSF), MIG, macrophage migration inhibitory factor (MIF), macrophage inhibitory protein (MIP)-1α, MIP-1β, nerve growth factor-beta (NGF-β), platelet-derived growth factor (PDGF-β), regulated-upon-activation normal T-cell expressed and presumably secreted (RANTES), stem cell factor (SCF), stem cell growth factor-beta (SCGF-β), stromal cell-derived factors 1-alpha (SDF-1α), TNF-α, TNF-β, TNF-related apoptosis-inducing ligand (TRAIL), and vascular endothelial growth factor (VEGF). A standard curve was used to determine cytokine concentrations in the samples using a 5-parameter logistic regression model. The inter- and intra-assay variability was performed by comparing cytokine concentrations in replicates plated on the same or across plates, with Spearman r values > 0.8 considered acceptable per cytokine. Cytokine levels below the lower limit of detection of the assay were reported as the mid-point between zero and the lowest concentrations measured for each cytokine. Data were presented as log10-transformed values to normalize distributions.

### 2.5. Assessment of Immune Cell Phenotype and Activation in Endocervical Cytobrushes 

**Figure 1:**
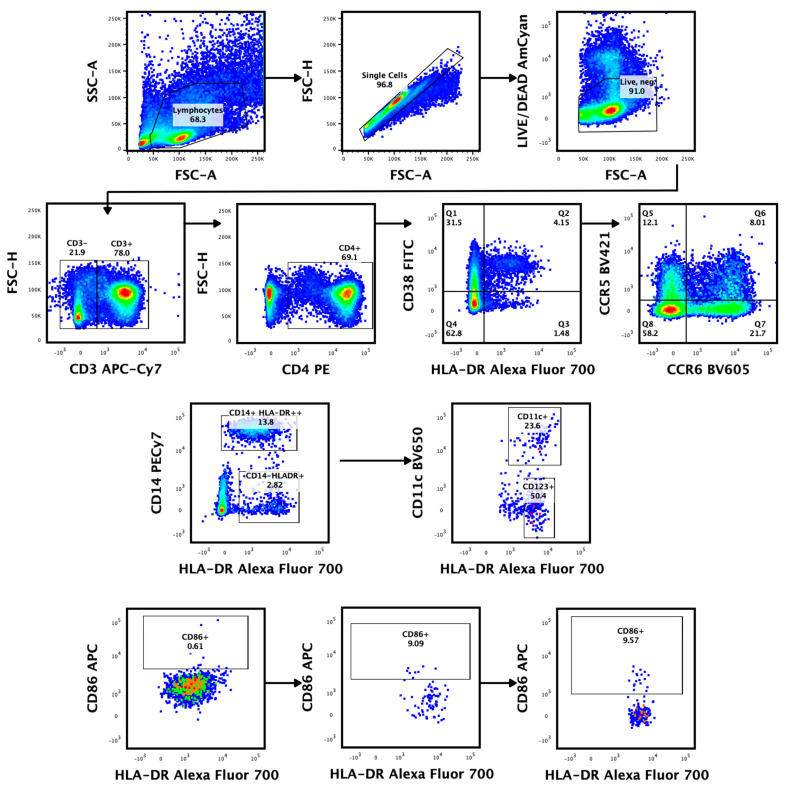
Identification of cervical T- and monocyte/dendritic-cell subsets from endocervical cytobrush specimens at three time points. Representative gating plot shows the exclusion of non-lymphocytes, doublets, dead (LIVE/DEAD™), CD19, and CD56 B lymphoid cells. The resulting cells were separated into CD3+ and CD3− subsets. The CD3+ T cells were further classified into CD4+ T cells. The expression of CD38, HLA-DR, CCR5, and CCR6 on CD4+ CD3+ lymphocytes was examined. The CD3- subset was separated into dendritic cell subsets: mDCs (HLADR+ CD14− CD11c+), pDCs (HLADR+ CD123+), and monocytes (HLADR+ CD14+ CD11c+). Activated DCs were identified using their CD86 expression, while monocytes were classified as CD14+ HLADR++.

Endocervical cytobrushes were collected as previously described using insertion and 360° rotation of a cytobrush in the endocervical os [32]. Cervical mononuclear cells were liberated from the brush through a combination of vortexing and washing; they were then filtered and processed for flow cytometry ex vivo, as previously described [33]. The cells were incubated with LIVE/DEAD Aqua fluorescence (Invitrogen from Thermo fisher scientific^TM^, Waltham, MA, USA) stain for 20 min at 4 °C in the dark, washed with FSC phosphate-buffer saline, and surface stained for 20 min at room temperature with following antibody cocktail: Alexa Fluor 700 (AF)-labelled anti-HLA-DR, APC-labelled anti-CD86, Brilliant Violet 785 (BV785)-labelled anti-CD16, Peridinin-chlorophyll-protein CY5.5 (PerCP-CY5.5)-labelled anti-CD123, BV650-labelled anti-CD11c (Biolegend, San Diego, CA, USA), Phycoerythrin (PE)-labelled anti-CD4, Allophycocynin-H7 labelled (APC-H7)-anti-CD3, Fluorescein Isothiocyanate (FITC)-labelled anti-CD38, BV605-labelled anti-CCR6, BV421-labelled anti-CCR5, PECy7-labelled anti-CD14, V510-labelled ant-CD19, and V510-labelled anti-CD56 (Becton Dickinson, Franklin Lakes, NJ, USA). Data acquisition was conducted using a FACSDiva Flow cytometer (Becton and Dickinson Immunocytometry Systems) and data were analyzed using FlowJo Software version 10 (Tree Star, Woodburn, OR, USA). Fluorescence minus one (FMO) staining was used to set gates to differentiate negative and positive populations [34]. All data were corrected for background, using the unstimulated condition. Figure 1 shows the gating strategy used to characterize cervical T and dendritic cells from cytobrush specimens.

### 2.6. Statistical Analysis

R software version 4.3.1 was used for statistical analysis. Continuous data were described using median and interquartile range (IQR), while categorical data were described using the number of participants (*n*) and percentage of participants with non-missing data. Wilcoxon rank-sum and Chi-square (or Fisher’s exact) tests were used to compare continuous and categorical data, respectively, in bivariate analysis. For paired data, the Wilcoxon signed-rank test was used. The baseline characteristics, clinical assessments, and sexual behaviors of the participants were compared across the outcome of BV infection after treatment. In addition, the clinical assessments, and sexual behaviors that varied over time were also compared across the outcomes at 6 and 12 weeks. Genital inflammation was classified as having 5 or more of 9 pro-inflammatory cytokines and chemokines (IL-1α, IL-6, IL-8, interferon gamma-induced protein (IP)-10, macrophage inflammatory protein (MIP)-1α, MIP-1β, monocyte chemotactic protein (MCP)-1, tumour necrosis factor (TNF)-α, and IL-1β) above the 75th percentile [35,36]. Cell population percentages between timepoints (baseline, 6 weeks, and 12 weeks) were compared using a Wilcoxon signed-rank test. A Wilcoxon rank-sum test was used to determine whether at 12 weeks the outcomes of BV after treatment were associated with cell population percentages. In this investigation, a significance level of 0.05 was employed to determine the statistical significance of the results. Given the exploratory nature of the analyses conducted in this paper, no adjustments were made for multiple comparisons. The findings presented herein are intended to generate hypotheses rather than provide confirmatory evidence.

## 3. Results

### 3.1. Participant Characteristics

Thirty-two women had 16S rRNA gene sequences, cytokine, and cellular data for all three visits. The cohort characteristics are presented in Table 1. The median age was 24 years (interquartile range (IQR) 21–28 years), and more than two-thirds reported condom use during the study (72%; 23/32). Only 6% (2/32) of these women reported consistent condom use, with the majority reporting that they used condoms occasionally (63%; 20/32). Only 47% (15/32) of study participants reported the use of any form of contraception, with the majority of these reporting the use of progesterone-based injectable contraceptives (32%; 10/32).

At baseline, 28% (9/32) of the women had evidence of genital inflammation, defined by having at least 5/9 inflammatory cytokines above the 75th percentile, as described previously [36,37]. Evidence of genital inflammation reduced slightly after treatment for BV, with 22% (7/32) of women having inflammation at 6 weeks and 19% (6/32) having inflammation at 12 weeks. All 32 women had Nugent scores ≥ 4 at baseline; the majority (63%; 20/32) were considered BV positive (Nugent score ≥ 7) and 38% (12/32) had intermediate Nugent scores (4–6). In addition, 78% (25/32) of women had any STI or vaginal candidiasis at baseline, with *C. trachomatis* being the most prevalent bacterial STI (34%, 11/32), while 16% (5/32) of women had Candidiasis evident. All women who had Nugent scores ≥ 4 at baseline and/or an STI received targeted antibiotic or antifungal treatment and were then tested for cure at their 6-week visit, and again at 3 months. At 6 weeks, only 25% (8/37) of women were BV negative and this proportion remained unchanged at 12 weeks.

### 3.2. Shifts in Cell Phenotypes Following MDZ Treatment 

**Figure 2:**
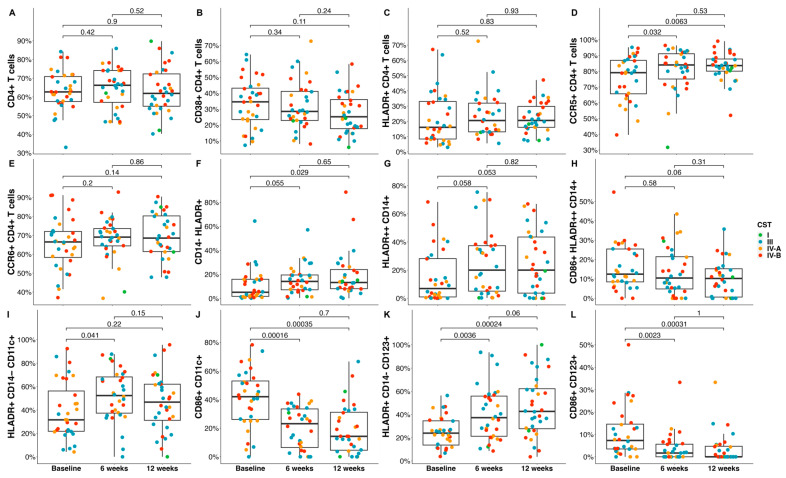
Frequency distribution of T- and dendritic-cell subsets in cytobrush specimens at baseline compared to 6- and 12-weeks post-treatment. Graph shows the differences over time for the frequencies of cervical (**A**) CD4+ T cells, (**B**) CD38+ CD4+ T cells, (**C**) HLADR+ CD4+ T cells, (**D**) CCR5+ CD4+ T cells, (**E**) CCR6+ CD4+ T cells, (**F**) CD14− HLADR+, (**G**) HLADR++ CD14+, (**H**) CD86+ HLADR++ CD14+, (**I**) HLDR+ CD14− CD11c+, (**J**) CD86+ CD11c+, (**K**) HLADR+ CD14− CD123+, and (**L**) CD86+ CD123+. Green color depicts CST I, blue CST III, orange CST IV-A, and red CST IV-B. The Mann–Whitney U test was used to compare the frequency distribution of T- and dendritic-cell subsets at pre- and post-treatment timepoints.

Here, we investigated changes in cervical immune cell profiles following metronidazole treatment ex vivo. The frequency of CCR5+ CD4+ T cells was significantly increased in cytobrush specimens collected at 6- (5%, *p* = 0.034) and 12-weeks (7%, *p* = 0.0096) post-treatment compared to the pre-treatment time point (Figure 2). In addition, the frequency of pDCs (HLADR+ CD14− CD123) was significantly increased at 6 (16%, *p* = 0.0036) and 12 weeks (22%, *p* = 0.0002), while mDCs (HLRDR+ CD14− CD11c+) were only increased at 6-weeks (13%, *p* = 0.0410) post-treatment compared to the pre-treatment timepoint. The frequency of CD14− HLDR+ was increased at 12-weeks (8%, *p* = 0.029) post-treatment. In contrast, the MDZ treatment reduced the frequencies of activated mDCs (CD86+ CD11c+, *p* = 0.00016; at 6 weeks: −21%, *p* = 0.0002; 12 weeks: −22%, *p*= 0.0004) and pDCs (CD86+ CD123+, at 6 weeks: −7%, *p* = 0.0023; 12 weeks: −7%, *p* = 0.0003) at the post-treatment compared to the pre-treatment timepoint. Furthermore, we observed a trend towards an increase in the frequency of monocytes (HLADR++ CD14+) at 6 (10%, *p* = 0.058) and 12 weeks (10%, *p* = 0.053), while contrasting trends in the frequency of activated monocytes (CD86+ HLRDR++ CD14+) were observed at 6-weeks (−2%, *p* = 0.06) post-treatment. 

We further tested whether genital inflammation increases the number of activated cells within the mucosa. Women with genital inflammation had increased frequencies of pDCs and mDCs compared to the non-inflamed group, after controlling for age and time in the study (Supplementary Table S1). We observed no significant differences in proportions of other T-cell and DC subsets among women with genital inflammation and the non-inflamed group.

### 3.3. Vaginal Microbiota Characteristics Pre- and Post-Metronidazole

Based on the composition and relative abundance of bacterial species, bacterial communities were assigned into four community state types: CST I dominated by *L. crispatus*, CST III dominated by *L. iners*, CST IV-A representing samples with high relative abundance of BVAB1, and CST IV-B characterized by *G. vaginalis* dominance. Among the vaginal samples of women with BV (Nugent score ≥ 4) at baseline, most were dominated by *G. vaginalis* (CST IV-B; 34%, 11/32) while *L. iners* (CST III, 34%, 11/32) was the most common lactobacilli. 

**Figure 3:**
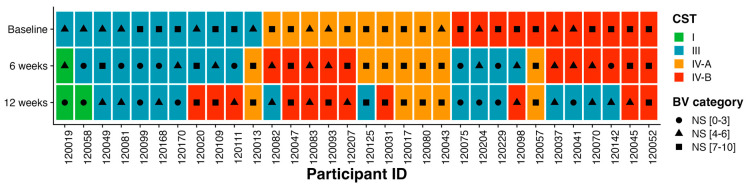
Composition of bacterial species and transitions within bacterial communities over the 12–week period. Box plots showing CSTs assigned to individual participants over 3 time points at baseline, 6 weeks, and 12 weeks. Green color depicts CST I, blue CST III, orange CST IV–A, and red CST IV–B.

Next, we modelled the movement of bacterial communities between BV-related CSTs (CST IV-A or IV-B) and *Lactobacillus*-related state types (CST I and III) in MDZ-treated women over time. More than half of the participants (53%, 17/32) remained in the same profile of BV-associated CSTs [CST IV-A (50%, 5/10) or CST IV-B (55%, 6/11)] at 6-weeks post MDZ treatment, but only 16% (5/32) remained in their baseline CST at 12 weeks (Figure 3). The proportions of participants transitioning from a profile of BV-associated CSTs IV-B into *Lactobacillus*-dominant CSTs III increased from 13% (4/32) at week 6 to 28% (9/32) at week 12. Similarly, the proportions of participants transitioning from *Lactobacillus*-dominated communities into BV-associated CSTs also increased from 3% (1/32) at 6 weeks to 13% (4/32) at 12 weeks. Although *L. crispatus* was rare pre-MDZ, only 1/32 (3%) woman had *L. crispatus*-dominant communities in week 6 and 12 post-treatment, while others only achieved this dominance at 12-weeks post-MDZ visits.

### 3.4. Effect of Bacterial Community Transition Status on Cell Percentage Difference

**Figure 4:**
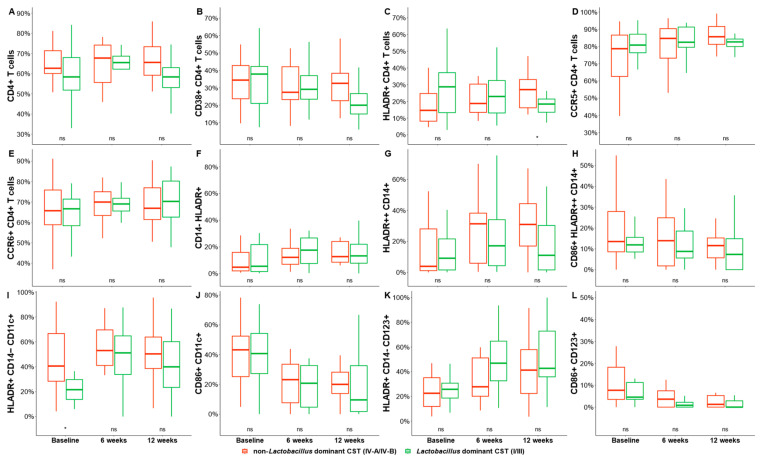
Frequencies of cervical T-cell and DC subsets in women with a cervicovaginal microbiota dominated by *Lactobacillus* spp. (predominantly *L. iners*, red) and non-*Lactobacillus* at baseline, 6 weeks, and 12 weeks after initiating MDZ. Graphs show the differences between pre- and post-MDZ initiation for the frequencies of cervical: Graph shows the differences over time for the frequencies of cervical (**A**) CD4+ T cells, (**B**) CD38+ CD4+ T cells, (**C**) HLADR+ CD4+ T cells, (**D**) CCR5+ CD4+ T cells, (**E**) CCR6+ CD4+ T cells, (**F**) CD14− HLADR+, (**G**) HLADR++ CD14+, (**H**) CD86+ HLADR++ CD14+, (**I**) HLDR+ CD14− CD11c+, (**J**) CD86+ CD11c+, (**K**) HLADR+ CD14− CD123+, and (**L**) CD86+ CD123+. Red color depicts non-*Lactobacillus* dominant CST (IV-A/IV-B) and red depicts *Lactobacillus* dominant CST (I/III). Statistical comparisons were performed using Mann–Whitney U tests for unmatched data or the Kruskal–Wallis test with false discovery rate correction for cross-sectional data. * Indicates significant differences (*p* < 0.05), while *ns* indicates non–significant difference (*p* > 0.05).

We hypothesized that shifts in the vaginal microbiota are key regulators of mucosal inflammatory responses, causing immune activation that increases the risk for HIV acquisition. A Mann–Whitney U test was used to examine the relationship between the vaginal microbiome and HIV-susceptible cell number by comparing cell percentage differences between *Lactobacillus*-dominant CSTs (combination of CST I and III) and BV-associated CSTs (CST IVA and IVB) over the three timepoints. The frequency of activated CD4+ T cells (HLADR+ CD4+, *p* = 0.0364) and mDCs (HLADR+ CD14− CD11c+, *p* = 0.0321) was significantly higher in women with highly diverse bacterial communities compared to those with a cervicovaginal microbiota dominated by *Lactobacillus* spp. (predominantly *L. iners*). We observed no significant changes in the frequency of any of the other cell phenotypes between women with a cervicovaginal microbiota dominated by *Lactobacillus* spp. compared to those with highly diverse bacterial communities (reminiscent of bacterial communities found in BV, Figure 4).

**Figure 5:**
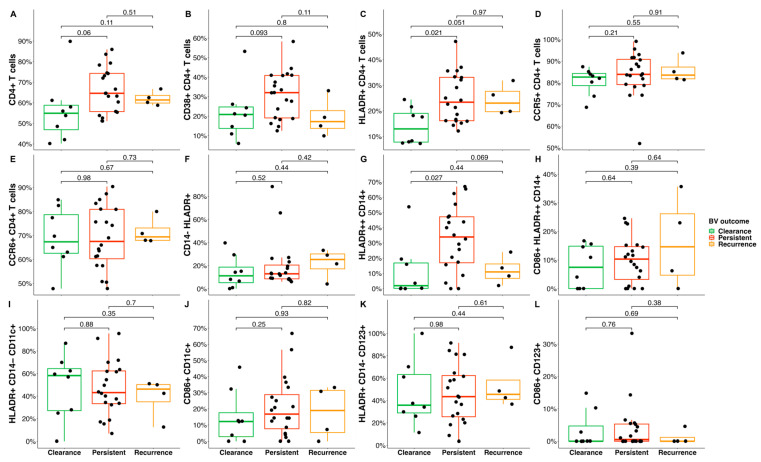
Frequencies of cervical T-cell and DC subsets in women who cleared BV (green) and compared to women with persistent or recurrent BV after initiating MDZ. Graph shows the differences over time for the frequencies of cervical (**A**) CD4+ T cells, (**B**) CD38+ CD4+ T cells, (**C**) HLADR+ CD4+ T cells, (**D**) CCR5+ CD4+ T cells, (**E**) CCR6+ CD4+ T cells, (**F**) CD14− HLADR+, (**G**) HLADR++ CD14+, (**H**) CD86+ HLADR++ CD14+, (**I**) HLDR+ CD14− CD11c+, (**J**) CD86+ CD11c+, (**K**) HLADR+ CD14− CD123+, and (**L**) CD86+ CD123+. Clearance is depicted in green, persistent in red, and recurrence in orange. Individual associations are shown between BV status and immune subsets including CD3+, CD4+ T cells, CD38+ CD4+ T cells, HLA-DR+ CD4+ T cells, CCR5+ CD4+ T cells, CCR6+ CD4+ T cells, CD14+, CD11c+ CD14+, and CD123+ CD14+ cells.

Next, a Mann–Whitney U test was used to compare the frequencies of cervical T-cell and DC subsets in women who cleared BV (at 12-weeks post-MDZ) to those who had persistent BV (at 12-weeks post-MDZ). We observed significantly higher frequencies of activated CD4 T cells (HLA-DR+ CD4+, *p* = 0.021, mean difference of 7%) and monocytes (HLADR++ CD14+, *p* = 0.027) in endocervical cytobrush specimens of women experiencing persistent BV relative to those who cleared BV (Figure 5). Although not significant, frequencies of CD4+ T (*p* = 0.060, mean difference of 9%) in women who experienced persistent BV trended higher compared to those who cleared BV. Furthermore, the frequency of CD38+ CD4+ T cells (*p* = 0.093, mean difference of 7%) showed a trend towards being elevated in women experiencing persistent BV compared to women who experienced BV recurrence.

We further assessed whether the frequency of T-cell and DC subsets was stable over time in women who had BV resolution or persistent at 12 weeks post-MDZ. There were no significant differences between the frequency of T-cell and DC subsets among women who cleared BV over time. The frequency of CCR5+ CD4+ T cells was significantly higher in women with persistent BV at 6- (*p* = 0.0058, mean difference of 2%) and 12-weeks (*p* = 0.0095, mean difference of 2%) post-MDZ compared to baseline (Supplementary Figure S1). Similarly, the frequency of HLADR (CD14− HLADR+) was significantly increased in women with persistent BV at 6- (*p* = 0.0034, mean difference of 2%) and 12-weeks (*p* = 0.0030, mean difference of 2%) post-MDZ compared to baseline. Monocytes (6 weeks: *p* = 0.0160, mean difference of 2%; 12 weeks: *p* = 0.0034, mean difference of 2%), pDCs (6 weeks: *p* = 0.0105, mean difference of 2%; 12-weeks: *p* = 0.0042, mean difference of 2%) and activated mDCs (6 weeks: *p* = 0.0084, mean difference of 2%; 12 weeks: *p* = 0.0106, mean difference of 2%) were also increased in women with persistent BV post-MDZ compared to pre-MDZ timepoint. Women with persistent BV had higher frequencies of activated pDCs (*p* = 0.0069, mean difference of 2%) and monocytes (*p* = 0.0239, mean difference of 2%) at 12-weeks post-MDZ compared to baseline. Women who cleared BV had decreased frequencies of CD38+ CD4+ T cells (*p* = 0.0423, mean difference of 1%) and HLADR+ CD4+ T cells (*p* = 0.0294, mean difference of 1%) at 12-weeks post-MDZ compared to baseline. The frequencies of activated mDCs (*p* = 0.0299, mean difference of 1%) were decreased at 6-weeks post-MDZ compared to baseline.

## 4. Discussion

The diverse non-*Lactobacillus* dominant communities are closely associated with elevated genital inflammation and an increased HIV risk, likely due to increasing the mucosal HIV target-cell frequency and T-cell activation [14,15]. However, less is known about the mucosal immune milieu pre- and post-MDZ treatment, including in those who cleared or had persistent BV. The current study determined the effects of MDZ treatment on the frequency of mucosal immune cell subsets in endocervical cytobrushes from women with a laboratory diagnosed STI and/or BV. We observed a significantly higher number of the activation marker HLA-DR and activated CCR5+ CD4+ T cells in cytobrush specimens post-MDZ treatment, and this was specifically observed in the BV-persistent subgroup. Similarly, the frequencies of DC subsets (pDCs and mDCs) were increased post-MDZ treatment. *L. iners* was the most common *Lactobacillus* species pre- and post-MDZ. Women classified as having persistent BV (Nugent score > 4) had significantly increased frequencies of activated CD4 T cells (HLA-DR+ CD4+ T) and monocytes (HLADR++ CD14+) compared to those who cleared BV (Nugent score < 3) at 12-weeks post-MDZ. 

While recent studies have demonstrated that MDZ has multiple effects on different components of immunity [9,15], very few have evaluated the association between the mucosal immune milieu pre- and post-BV treatment [38]. We observed a significantly increased frequency of endocervical CCR5+ CD4+ T cells and pDCs (HLADR+ CD14− CD123+) in cytobrush specimens collected at 6- and 12-weeks post-treatment compared to the pre-treatment timepoint. CCR5-expressing T lymphocytes are densely populated in the subepithelial stromal tissues in the vagina and CCR5 is a key cellular co-receptor required for HIV entry into CD4 T lymphocytes, including cell-to-cell spread [38]. DCs are known to play an important role in the host response to infection and are the first cells to sense and respond to microbes [39]. DCs interact with the microbes via pathogen-associated molecular patterns to trigger specific T- and B-cell responses [40,41,42]. The increased levels of CCR5+ cells and pDCs may be attributed to the persistence of BV-associated bacteria and this is confirmed through increased levels over time in women who had persistent BV. These findings suggest that the putative mechanism mediating HIV susceptibility in women with persistent BV is through a DC–microbe interaction and the recruitment of activated CCR5+ cells, which are known to facilitate HIV acquisition by allowing the virus to attach to the cell and establish an infection.

*L. iners* is normally the first *Lactobacillus* spp. to emerge after treatment of BV with metronidazole. Although *L. crispatus* has been associated with reproductive health benefits (including a decrease in pre-term births, STIs, genital inflammation, and HIV risk), mounting evidence suggests that *L. iners* may contribute to the onset and maintenance of BV or intermediate-BV status [43,44]. Furthermore, using a Markov model, we [9] and others [45,46] have previously demonstrated that *L. iners* dominant communities have a high probability of transitioning to BV-associated states. Consistent with previous studies, the current study also observed a high prevalence of *L. iners* and near-pan absence of *L. crispatus* after MDZ treatment. It has been reported that the standard BV treatment tends to promote dominance by *L. iners* rather than *L. crispatus*, which may partially explain the high recurrence rates and long-term vaginal dysbiosis, especially after repeated treatment cycles [13,47,48]. There are many knowledge gaps remaining regarding the activity and role of *L. iners* in the vaginal microbiota, including whether this enigmatic bacterium is only a predictive biomarker of the transition in vaginal ecology or a risk factor for BV and its impact on the mucosal immune microenvironment. Due to the important role non-*iners Lactobacillus* spp. play in maintaining the optimal mucosal environment, the discovery of interventions that shift *L. iners*-dominant communities to *L. crispatus*-dominant communities, along with novel approaches to selectively inhibit *L. iners* while sparing protective vaginal lactobacilli, is needed and may potentially provide insights into bacterial community vulnerabilities that can translate into new therapeutic approaches. 

Although multiple studies have explored the association between vaginal microbiota and mucosal HIV target-cell frequency and activation [9,14,15,18], very few have evaluated the relative effects of BV eradication or recurrence on the mucosal immune milieu. We previously showed that BV clearance reduced levels of pro-inflammatory cytokines (TNF-α, IL-1β, IL-8, and LIF), while BV persistence was associated with the increased concentrations of cytokines (IL-1α, IL-18, MIF, IL-7, and LIF) linked to an increased risk of acquiring HIV infection. These findings, however, were in the absence of testing the effect on frequency and activation of HIV-susceptible cells [9]. In the current study, BV persistence was associated with a significant increase in frequencies of activated CD4 T cells and monocytes compared to women who cleared BV at 12 weeks. These findings are in agreement with previous studies that showed an increased frequency of activated CD4+ T cells in women with highly diverse non-*Lactobacillus*-dominant communities [14,15]. In addition, we also found that increased frequencies of activated CD4 T cells and mDCs were significantly associated with the highly diverse bacterial communities usually present in BV. Furthermore, we observed increased levels of HLA-DR over time in women who had persistent BV. HLA-DR is an antigen-presenting molecule that is typically expressed at high levels on professional antigen-presenting cells [49], but is also expressed on chronically activated T cells [50,51,52]. HLA-DR expression is associated with immune checkpoint molecules expressed on cells susceptible to HIV infection, such as percentages of activated CD4+ T cells [53]. In contrast, women who cleared BV over time had decreased levels of activated CD4 T cells (CD38+ CD4+ T cells and HLADR+ CD4+ T cells) and activated mDCs (CD84+ CD11c+). A highly diverse and strictly anaerobic vaginal microbiota in women of African descent has been associated with increased genital inflammation and HIV risk, likely due to an increased activation and recruitment of HIV-susceptible cells to the vaginal mucosa [7,14,15,16]. These findings suggest that diverse microbial communities may influence HIV susceptibility by increasing the frequency and differentiation of mucosal HIV target cells within the genital mucosa.

The limitations of our study include a small sample size that restricted the ability to assess in detail the effects of BV clearance, persistence, or recurrence on the mucosal immune milieu. Another limitation was the lack of a control group of BV-negative women to investigate immune subset changes between the MDZ experienced and naïve groups. Additionally, we did not investigate the impact of other potential co-factors such as STIs (bacterial and viral), hormonal contraceptives, diet, hormonal status, and other vaginal disorders (e.g., aerobic vaginitis) on microbiota, although each may affect the immune milieu. A major strength of our study is that we leveraged the existing inflammatory and 16S rRNA data to longitudinally assess the impact of BV treatment outcomes on the frequency of mucosal immune subsets.

## 5. Conclusions

*L. iners* was the most predominant *Lactobacillus* spp. before and after treatment with metronidazole. Treatment failure and persistent BV contributed to an increase in the cell-associated inflammatory response, potentially as a result of exposure to persistent bacterial diversity that is consistently associated with elevated genital inflammation and increased HIV risk. The current treatment regimens for BV are less likely to promote the absolute abundance of low-inflammatory *L. crispatus* communities. Therefore, viable alternative strategies such as pre- and pro-biotics, vaginal microbiome transplantation, and phage endolysins to improve *Lactobacillus* representation are proposed as part of the curative or preventative vaginal dysbiosis interventions.

## Figures and Tables

**Table 1:** Demographic, clinical, and behavioral data of study population.

Variable	Level	Baseline	6 Weeks	12 Weeks
N		32	-	-
Age, median [IQR]		24 [21, 28]	-	-
Condom use, *n* (%)	No	9 (28.1)	-	-
	Yes	23 (71.9)	-	-
Frequent use of condom, *n* (%)	Always	2 (6.2)	-	-
	Sometimes	20 (62.5)	-	-
	Never	10 (31.2)	-	-
Contraceptive use, *n* (%)	No	17 (53.1)	-	-
	Yes	15 (46.9)	-	-
Contraception type, *n* (%)	Oral	1 (3.1)	-	-
	Injectable	10 (31.2)	-	-
	Implant	4 (12.5)	-	-
	None	17 (53.1)	-	-
BV score category, *n* (%)	Positive	20 (62.5)	13 (40.6)	12 (37.5)
	Intermediate	12 (37.5)	11 (34.4)	12 (37.5)
	Negative	0 (0.0)	8 (25.0)	8 (25.0)
BV community state type, *n* (%)	I	0 (0.0)	1 (3.1)	2 (6.2)
	III	11 (34.4)	13 (40.6)	14 (43.8)
	IV-B	11 (34.4)	11 (34.4)	11 (34.4)
	IV-A	10 (31.2)	7 (21.9)	5 (15.6)
Genital inflammation, *n* (%)	No	23 (71.9)	25 (78.1)	26 (81.2)
	Yes	9 (28.1)	7 (21.9)	6 (18.8)
Previous sexual activity, *n* (%)	No	25 (78.1)	22 (71.0)	20 (74.1)
	Yes	7 (21.9)	9 (29.0)	7 (25.9)
Candidiasis, *n* (%)	No	27 (84.4)	28 (87.5)	26 (81.2)
	Yes	5 (15.6)	4 (12.5)	6 (18.8)
Any STI, *n* (%)	No	12 (37.5)	26 (81.2)	24 (75.0)
	Yes	20 (62.5)	6 (18.8)	8 (25.0)
*Trichomoniasis*, *n* (%)	Negative	28 (87.5)	31 (96.9)	32 (100.0)
	Positive	4 (12.5)	1 (3.1)	0 (0.0)
*Gonorrhea*, *n* (%)	Negative	28 (87.5)	32 (100.0)	31 (96.9)
	Positive	4 (12.5)	0 (0.0)	1 (3.1)
*Chlamydia*, *n* (%)	Negative	21 (65.6)	31 (96.9)	32 (100.0)
	Positive	11 (34.4)	1 (3.1)	0 (0.0)
Herpes simplex virus type 2, *n* (%)	Negative	31 (96.9)	31 (96.9)	31 (96.9)
	Positive	1 (3.1)	1 (3.1)	1 (3.1)

## Data Availability

The data that support the findings of this study are available on request from the corresponding author (S.N.).

## Supplementary Materials

The following supporting information can be downloaded at: https://www.mdpi.com/article/10.3390/microorganisms11112643/s1, Figure S1: Comparison of baseline cellular percentages to the preceding weeks within BV outcomes (clearance in green; persistence in red and recurrence in orange); Table S1: Frequency distribution of T and dendritic cell subsets in women who had genital inflammation.
