# Supplementary material for: Metronidazole Treatment Failure and Persistent BV Lead to Increased Frequencies of Activated T- and Dendritic-Cell Subsets
Source: Microorganisms. 2023 Oct 27;11(11):2643. doi: 10.3390/microorganisms11112643 (PMC10673474; doi:10.3390/microorganisms11112643)
Supplement: Supplementary file 1 [file microorganisms-11-02643-s001.zip › Supplementary Figure S1.pdf]

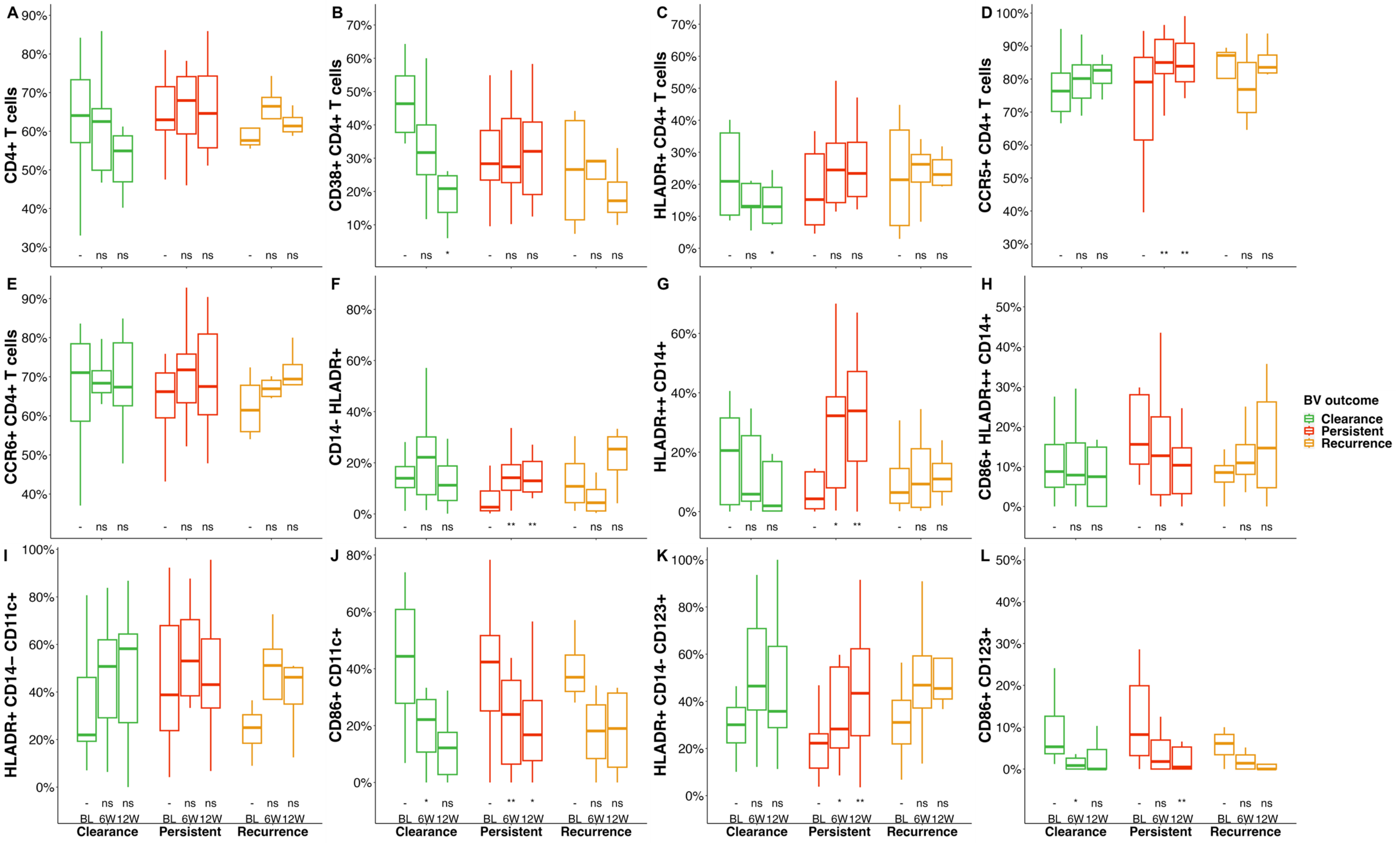

Supplementary Figure S1. Comparison of baseline cellular percentages to the preceding weeks within BV outcomes (clearance in green; persistence in red and recurrence in orange). Graph shows the differences over time for the frequencies of cervical (**A**) CD4+ T cells, (**B**) CD38+ CD4+ T cells, (**C**) HLADR+ CD4+ T cells, (**D**) CCR5+ CD4+ T cells, (**E**) CCR6+ CD4+ T cells, (**F**) CD14- HLADR+, (**G**) HLADR++ CD14+, (**H**) CD86+ HLADR++ CD14+, (**I**) HLDR+ CD14- CD11c+, (**J**) CD86+ CD11c+, (**K**) HLADR+ CD14- CD123+, and (**L**) CD86+ CD123+. Clearance is depicted in green, persistent in red, and recurrence in orange. * Indicates significant differences of p <0.05, ** indicates significant differences p < 0.01 and *ns* indicates non–significant difference (p >0.05).
