# Supplementary material for: Metronidazole Treatment Failure and Persistent BV Lead to Increased Frequencies of Activated T- and Dendritic-Cell Subsets
Source: Microorganisms. 2023 Oct 27;11(11):2643. doi: 10.3390/microorganisms11112643 (PMC10673474; doi:10.3390/microorganisms11112643)
Supplement: Supplementary file 1 [file microorganisms-11-02643-s001.zip › Supplementary Table S1.pdf]

Supplementary Table S1. Frequency distribution of T and dendritic cell subsets in women who had genital inflammation

Cell Phenotype	Univariable						Multivariable (adjusted for age and time in study)					
			95% CI						95% CI			
	β coefficient	SE	Lower	upper	P-value	FDR	Adj β coefficient	SE	Lower	Upper	Adj P-value	FDR
CD4+ T cells	0.008	0.026	-0.048	0.064	0.774	0.8716	0.013	0.028	-0.048	0.074	0.652	0.8424
CD38+ CD4+ T cells	0.052	0.034	-0.021	0.124	0.147	0.666	0.044	0.036	-0.035	0.123	0.25	0.765
HLADR+ CD4+ T cells	0.017	0.032	-0.052	0.087	0.597	0.8716	0.005	0.035	-0.072	0.082	0.884	0.945
CCR5+ CD4+ T cells	-0.03	0.028	-0.09	0.031	0.313	0.666	-0.037	0.031	-0.104	0.03	0.255	0.765
CCR6+ CD4+ T cells	-0.03	0.029	-0.091	0.032	0.321	0.666	-0.032	0.031	-0.099	0.035	0.321	0.7704
CD14- HLADR+	-0.009	0.035	-0.085	0.067	0.799	0.8716	0.018	0.034	-0.056	0.091	0.608	0.8424
HLADR++ CD14+	0.007	0.053	-0.108	0.122	0.901	0.901	0.022	0.055	-0.099	0.143	0.702	0.8424
CD86+ HLADR++ CD14+	-0.033	0.026	-0.09	0.025	0.241	0.666	-0.022	0.028	-0.083	0.039	0.451	0.7971
HLADR+ CD14- CD11c+	0.057	0.057	-0.065	0.179	0.333	0.666	0.126	0.057	0.002	0.249	0.046	0.276
CD86+ CD11c+	0.016	0.043	-0.077	0.109	0.719	0.8716	0.035	0.046	-0.066	0.135	0.465	0.7971
HLADR+ CD14- CD123+	-0.101	0.05	-0.209	0.006	0.063	0.666	-0.149	0.05	-0.259	-0.04	0.012	0.144
CD86+ CD123+	0.012	0.029	-0.054	0.079	0.684	0.8716	-0.002	0.034	-0.086	0.081	0.945	0.945
